# Association of Methylenetetrahydrofolate Dehydrogenase 1 Polymorphisms with Cancer: A Meta-Analysis

**DOI:** 10.1371/journal.pone.0069366

**Published:** 2013-07-19

**Authors:** Hongtuan Zhang, Hui Ma, Liang Li, Zhihong Zhang, Yong Xu

**Affiliations:** 1 National Key Clinical Specialty of Urology, Second Affiliated Hospital of Tianjin Medical University, Tianjin Key Institute of Urology, Tianjin, China; 2 Department of Gynaecology and Obstetrics, second hospital of Tianjin Medical University, Tianjin Medical University, Tianjin, China; 3 Laboratory of Population and Quantitative Genetics, School of Life Sciences, Tianjin Medical University, Tianjin, China; MOE Key Laboratory of Environment and Health, School of Public Health, Tongji Medical College, Huazhong University of Science and Technology, China

## Abstract

**Background:**

Studies investigating the association between single-nucleotide polymorphisms (SNPs) of the methylenetetrahydrofolate dehydrogenase 1 (MTHFD1) and cancer risk report conflicting results. To derive a more precise estimation of the relationship between MTHFD1 polymorphisms and cancer risk, the present meta-analysis was carried out.

**Methodology/Principal Findings:**

A comprehensive search was conducted to determine all the eligible studies about MTHFD1 polymorphisms and cancer risk. Combined odds ratios (ORs) and 95% confidence intervals (CIs) were used to assess the strength of the association between the MTHFD1 polymorphisms and cancer risk. We investigated by meta-analysis the effects of 2 polymorphisms in MTHFD1: G1958A (17 studies, 12348 cases, 44132 controls) and G401A (20 studies, 8446 cases, 14020 controls). The overall results indicated no major influence of these 2 polymorphisms on cancer risk. For G1958A, a decreased cancer risk was found in acute lymphoblastic leukemia (ALL)/Asians (the dominant: OR = 0.74, 95% CI = 0.58–0.94, P = 0.01; allelic: OR = 0.80, 95% CI = 0.65–0.99, P = 0.04) and other cancers (recessive: OR = 0.80, 95% CI = 0.66–0.96, P = 0.02). For G401A, the data showed that MTHFD1 G401A polymorphism was associated with a decreased colon cancer risk under dominant model (OR = 0.89, 95% CI = 0.80–0.99, P = 0.04).

**Conclusions:**

The results suggest that MTHFD1 G1958A polymorphism might be associated with a decreased risk of ALL and other cancers. Meanwhile, the MTHFD1 G401A might play a protective role in the development of colon cancer. Large-scale and well-designed case-control studies are necessary to validate the risk identified in the present meta-analysis.

## Introduction

Cancer remains a major public health problem in the world. The complex etiology of this disease is not yet fully elucidated. Identifying risk factors for cancer is critically important to develop potential interventions and to expand our understanding of the biology of this disease. Cancer is a disease resulting from complex interactions between environmental and genetic factors [1]–[3]. An increasing number of cancers also involve an infectious agent. Infection with the bacterium Helicobacter pylori predisposes to gastric cancer. Hepatitis B virus and hepatitis C virus are associated with liver cancer, and Epstein-Barr virus is associated with lymphoma and nasopharyngeal cancer. Human papillomavirus is a major cause of cervical, anal, penile, and oropharyngeal cancer. It is well known that the number of infection-related cancers is estimated at nearly 2 million cases per year, accounting for almost 20% of all cancer case. Genetic factors, including sequence alterations and organization aberrations of the cellular genome that range from single-nucleotide substitutions to gross chromosomal changes, could modulate several important biological activities and cancer susceptibility. Single nucleotide polymorphisms (SNPs) are the most common source of human genetic variation, and they may contribute to an individual’s susceptibility to cancer. In an attempt to clarify the exact mechanism by which genetic variation influences an individual’s susceptibility to cancer, many extensive studies have been performed worldwide.

Folate is a water-soluble B vitamin involved in one-carbon metabolism that plays an essential role in the synthesis, repair, and methylation of DNA [4]–[6]. Three main molecular mechanisms that link folate deficiency to tumor formation have been proposed. Folate deficiency can decrease global DNA methylation, which is associated with genetic instability and tumor formation. The second potential tumorigenic pathway of folate deficiency is increased uracil misincorporation during DNA replication. The third potential tumorigenic pathway of folate deficiency is enzymatic cytosine deamination at sites of DNA methylation. Folate metabolism provides one-carbon units necessary for the synthesis of nucleic acid bases and enables the conversion of methionine into S-adenosylmethionine (SAM), via its ability to methylate homocysteine. SAM is the universal methyl group donor in the majority of biochemical reactions including DNA methylation [7], [8]. If folate availability is continuously limited, an uncontrolled repair cycle can cause frequent breaks in DNA molecules and chromosomal damage [9]. All of these mechanisms contribute to genetic instability and may facilitate carcinogenesis, thus leading to the hypothesis that imbalances in folate metabolism can influence cancer risk.

MTHFD1 is the NADP-dependent tri-functional enzyme acting as 5,10-methylenetetrahydrofolate dehydrogenase; 5,10-methenyltetrahydrofolate cyclohydrolase; and 10-formylotetrahydrofolate synthetase [10]. MTHFD1, in three sequential reactions, provides 1-carbon derivatives of tetrahydrofolate that are substrates for biosynthesis of thymidylate, purinenucleotides, and methionine [10], [11]. Methionine is formed duringmethylation of homocysteine by methionine synthase, which uses methyl-tetrahydrofolate as a methyl donor [12]. Methionine adenosyltransferase, using methionine and ATP, induces formation of SAM [13]. Several polymorphisms in the MTHFD1 gene have been reported, including two common SNPs: G1958A (R653Q, rs2236225) and G401A (R134K, rs1950902). The MTHFD1 G1958A polymorphism is located within the 10-formyltetrahydrofolate synthetase domain and may modulate biosynthesis of thymidylate, purine nucleotides, and methionine effecting DNA methylation [11], [14], [15], [16]. The G401A SNP changes an arginine to a lysine in the dehydrogenase/cyclohydrolase domain of MTHFD1 and may affect these activities. However, no studies have investigated the functional consequences of this SNP.

A variety of molecular epidemiological studies have focused on the associations between MTHFD1 polymorphisms and cancer susceptibility. These studies have shown that the MTHFD1 polymorphisms occur in different types of cancer, but the results are inconclusive, partially because of the possible small effect of the polymorphism on cancer risk and the relatively small sample size in each of published studies. To solve the problem of inadequate statistical power and controversial results, it is necessary to carry out a systematic review and meta-analysis including subgroup analysis from all eligible studies to assess the association of the MTHFD1 polymorphisms with cancer risk.

## Materials and Methods

### Identification and Eligibility of Relevant Studies

We searched for articles using the terms “methylenetetrahydrofolate dehydrogenase 1” or “MTHFD1”, “polymorphism” or “variation”, and “cancer” or “carcinoma” or “neoplasm” or “malignance” in PubMed, Cochrane Library and Embase electronic databases, and all eligible studies were published up to February 20, 2013. We evaluated all the retrieved publications to retain the most eligible studies. Authors were contacted directly regarding crucial data not reported in original articles. Of the studies with the same or overlapping data by the same investigators, we selected the most recent ones with the most subjects. We evaluated all associated publications to retrieve the most eligible literatures. The reference lists of reviews and retrieved articles were hand searched at the same time. We did not include abstracts or unpublished reports. When overlapping data of the same patient population were included in more than one publication, only the most recent or complete study was used in this meta-analysis.

### Inclusion and Exclusion Criteria

The following inclusion criteria were used to select literatures for the meta-analysis: (1) information on the evaluating of MTHFD1 polymorphisms and cancer susceptibility; (2) only the case-control studies were considered; (3) controls were without cancer; and (4) sufficient genotype data were presented to calculate the odds ratio (OR) with 95% confidence interval (CI). In addition, the following exclusion criteria were also used: (1) repeated or overlapping studies; (2) no usable data reported; (3) animal studies; (4) control population including malignant tumor patients; and (5) the study only involved a case population.

### Data Extraction

Two investigators reviewed and extracted information from all eligible publications independently, according to the inclusion and exclusion criteria listed above. An agreement was reached by discussion between the two reviewers whenever there was a conflict. The following data were extracted: the first author’s last name, year of publication, ethnicity of the subjects, cancer type, and genotype distribution in cancer cases and controls. Different descents were categorized as Asian and Caucasian. If the ethnicity was not reported, we considered the ethnicity of the source population of the country where the study was performed. For case-control studies, data were extracted separately for each group whenever possible.

### Statistical Analysis

All statistical tests performed in this study were two-tailed and p values less than 0.05 were considered significant, unless otherwise stated. Statistical analyses were performed with Review Manage, version 5.0 and Stata 10.0. We assessed the departure from the Hardy-Weinberg equilibrium (HWE) for the control group in each study using an online HWE calculator (http://ihg.gsf.de/cgi-bin/hw/hwa1.pl).

The strength of the association between MTHFD1 G1958A polymorphism and cancer risk was measured by ORs, whereas a sense of the precision of the estimate was given by 95% Cls. We examined MTHFD1 G1958A genotypes using additive (AA vs GG), recessive (AA vs GA+GG), dominant (AA+AG vs GG), and allelic (A vs G) models. For G401A polymorphism, we evaluated the same effects. The significance of the pooled OR was determined by the Z-test and P<0.05 was considered statistically significant. Subgroup analysis was performed using stratification by cancer type and ethnicity, respectively.

If a cancer type contained less than two independent individual studies, it was categorized into the “other cancers” group. Testing for heterogeneity among studies was performed by Q-test. A P≥0.10 for the Q-test indicated a lack of heterogeneity among the studies. Either a random-effects model or fixed-effects model was used to calculate pooled effect estimates in the presence or absence of heterogeneity [17], [18], respectively. Additionally, we conducted sensitivity analyses by excluding each study individually and recalculating the ORs and 95% CI. An asymmetric plot indicates a possible publication bias. The symmetry of the funnel plot was further evaluated by Egger’s linear regression test. P<0.05 was considered statistically significant in publication bias.

## Results

### Characteristics of Studies

The process of selection of studies for inclusion in the meta-analysis is summarized in Figure S1. The database search identified 63 potentially relevant citations, of which 47 were judged to be of potential interest on the basis of the title. On the basis of the abstract, 39 studies were reviewed in their entirety. During the extraction of data, 17 articles were excluded, because they did not provide sufficient data needed for OR calculation, evaluating other polymorphisms of MTHFD1 and cancers, were review articles or their contents associated with cancer prognosis and therapy, leaving 22 articles [19]–[40]. In addition, of the studies with overlapping data [19]–[21], we selected the ones with the largest number of subjects [21]. 1 publication provided more than one individual study [39]. Finally, the pool of eligible studies included 37 studies [21]–[40], among which 17 with 12348 cases and 44132 controls were for G1958A polymorphism and 20 with 8446 cases and 14020 controls for G401A polymorphism. For the 37 case-control studies, baseline characteristics of the patients and control subjects were summarized, HWE in particular was assessed. 37 independent studies consisted of 2 Asians and 35 Caucasians. The genotype distributions among the controls of all studies were in agreement with HWE for all except 1 study [32]. Table S1 lists the main characteristics of these data sets about these two polymorphisms.

### Meta-analysis

The main results of the meta-analysis of the association between MTHFD1 polymorphisms and cancer risk are shown in Table 1. We first analyzed the association in the overall population. Then in order to obtain the exact consequence of the relationship between MTHFD1 polymorphisms and cancer susceptibility, stratified analyses by ethnicity and cancer type were performed. When the Q-test of heterogeneity was not significant, we conducted analyses using the fixed effect models. The random effect models were conducted when we detected significant between-study heterogeneity.

**Table 1:** Meta-Analysis of MTHFD1 Gene Polymorphisms and Cancer.

Genetic model	Sample size		Egger’s test	Test of association		Analysis model	Heterogeneity
(No. of studies)	Case	Control	P value	OR (95% CI)	P		P value
**rs2236225** Overall(17)							
AA vs GG	6314	22825	0.269	0.98 (0.92–1.05)	0.56	F	0.52
AA vs AG+GG	12348	44132	0.676	0.98 (0.92–1.04)	0.49	F	0.35
AA+AG vs GG	12348	44132	0.026	0.98 (0.94–1.03)	0.54	F	0.56
A vs G	24696	88264	0.074	0.99 (0.96–1.02)	0.43	F	0.35
Caucasian (15)							
AA vs GG	5934	22485		0.98 (0.92–1.05)	0.60	F	0.40
AA vs AG+GG	11802	43588		0.98 (0.92–1.04)	0.50	F	0.24
AA+AG vs GG	11802	43588		1.00 (0.95–1.05)	0.90	F	0.83
A vs G	23604	87176		0.99 (0.96–1.03)	0.64	F	0.49
Asian/ALL (2)							
AA vs GG	380	340		0.85 (0.48–1.52)	0.58	F	0.79
AA vs AG+GG	546	544		0.95 (0.54–1.69)	0.87	F	0.76
AA+AG vs GG	546	544		0.74 (0.58–0.94)	0.01	F	0.83
A vs G	1092	1088		0.80 (0.65–0.99)	0.04	F	0.71
Colorectal cancer (3)							
AA vs GG	1376	2203		0.97 (0.84–1.11)	0.62	F	0.27
AA vs AG+GG	2656	4256		0.98 (0.86–1.10)	0.69	F	0.45
AA+AG vs GG	2656	4256		0.98 (0.88–1.09)	0.67	F	0.35
A vs G	5312	8512		0.98 (0.92–1.05)	0.61	F	0.25
Head/neck cancer (2)							
AA vs GG	224	375		1.21 (0.85–1.71)	0.29	F	0.38
AA vs AG+GG	403	740		1.31 (0.96–1.79)	0.09	F	0.25
AA+AG vs GG	403	740		0.96 (0.74–1.24)	0.75	F	0.85
A vs G	806	1480		1.06 (0.89–1.27)	0.48	F	0.40
Prostate cancer (6)							
AA vs GG	3739	19077		1.00 (0.92–1.09)	0.91	F	0.87
AA vs AG+GG	7493	36941		1.00 (0.93–1.07)	0.94	F	0.83
AA+AG vs GG	7493	36941		1.01 (0.95–1.08)	0.69	F	0.75
A vs G	14986	73882		1.00 (0.96–1.05)	0.83	F	0.87
Other cancers (4)							
AA vs GG	595	830		0.81 (0.65–1.01)	0.06	F	0.17
AA vs AG+GG	1250	1651		0.80 (0.66–0.96)	0.02	F	0.19
AA+AG vs GG	1250	1651		0.95 (0.80–1.13)	0.56	F	0.34
A vs G	2500	3302		0.91 (0.82–1.01)	0.08	F	0.20
**rs1950902** Overall (20)							
AA vs GG	5981	9816	0.700	0.93 (0.79–1.08)	0.33	F	0.77
AA vs AG+GG	8446	14020	0.613	0.94 (0.80–1.09)	0.40	F	0.85
AA+AG vs GG	8446	14020	0.556	0.97 (0.89–1.05)	0.39	R	0.04
A vs G	16892	28040	0.669	0.97 (0.91–1.03)	0.33	R	0.06
Colon cancer (2)							
AA vs GG	1725	2663		0.91 (0.68–1.21)	0.51	F	0.66
AA vs AG+GG	2386	3808		0.94 (0.71–1.26)	0.68	F	0.56
AA+AG vs GG	2386	3808		0.89 (0.80–0.99)	0.04	F	0.36
A vs G	4772	7616		0.91 (0.83–1.00)	0.06	F	0.54
Other cancers (2)							
AA vs GG	604	688		0.86 (0.50–1.46)	0.57	F	0.83
AA vs AG+GG	865	936		0.82 (0.48–1.39)	0.46	F	0.96
AA+AG vs GG	865	936		1.13 (0.73–1.75)	0.59	R	0.03
A vs G	1730	1872		1.07 (0.77–1.50)	0.68	R	0.06

OR, odds ratio; CI, confidence interval.

### Quantitative Data Synthesis

#### G1958A

17 independent studies with a total of 12348 cases and 44132 controls were included in the meta-analysis for G1958A polymorphism. The Q-test of heterogeneity was not significant and we conducted analyses using fixed effect models in both overall and subgroup analyses. In overall population analyses, we did not find any associations between G1958A polymorphism and cancer susceptibility in any genetic model. In subgroup analysis by cancer type, no significant association with cancer risk was demonstrated in overall population with head and neck cancer (laryngeal cancer included), colorectal cancer, and prostate cancer. We can find that the population of subgroup of ALL is all Asians. For acute lymphoblastic leukemia, significantly decreased risk was observed in dominant model (OR = 0.74, 95% CI = 0.58–0.94, P = 0.01), and allelic model (OR = 0.80, 95% CI = 0.65–0.99, P = 0.04). With respect to other cancers, the results indicated that G1958A was significantly associated with a decreased other cancers risk under recessive model (OR = 0.80, 95% CI = 0.66–0.96, P = 0.02). G1958A polymorphism was significantly associated with decreased acute lymphoblastic leukemia risk under dominant model (OR = 0.74, 95% CI = 0.58–0.94, P = 0.01), and allelic contrast (OR = 0.80, 95% CI = 0.65–0.99, P = 0.04) in Asians.

#### G401A

There are 20 studies (8446 cases and 14020 controls) analyzing the relation between G401A polymorphism and the risk of cancer. In overall population, the Q test of heterogeneity was not significant and we conducted analyses using fixed effect models except in dominant model and allelic model. After subgroup analyses by cancer type, significant heterogeneity was effectively removed in colon cancer. We did not detect the association between G401A polymorphism and cancer risk in overall analysis. In subgroup analyses stratified by cancer type, the data suggested that G401A was associated with a decreased colon cancer risk under dominant model (OR = 0.89, 95% CI = 0.80–0.99, P = 0.04).

### Sensitivity Analysis

In order to compare the difference and evaluate the sensitivity of the meta-analyses, we conducted sensitivity analysis to evaluate the stability of the meta-analysis. A single study involved in the meta-analysis was deleted each time the analysis was performed to reflect the influence of the individual data set on the pooled ORs. The corresponding pooled ORs were not materially altered (data not shown). Hence, results of the sensitivity analysis suggest that the data in this meta-analysis are relatively stable and credible.

### Publication Bias

Publication bias was assessed by visual inspection of funnel plots in which the standard error of the log (OR) of each study was plotted against its log (OR). An asymmetric plot suggests a possible publication bias. Funnel plot asymmetry was assessed by the method of Egger’s linear regression test, a linear regression approach for measuring funnel plot asymmetry on the natural logarithm scale of the OR. As a result, publication bias was identified in certain comparisons (G1958A: AA+AG vs GG). The detailed data were shown in Table 1.

## Discussion

SNP is the most common form of human genetic variation, and may contribute to individual’s susceptibility to cancer. However, the underlying molecular mechanism is unknown. Genetic polymorphisms in the folate pathway have been investigated in a wide variety of diseases, such as neural tube defects [41], pancreatic cancer [42], and congenital heart defects [43]. In recent years, many studies have been conducted to investigate the association between MTHFD1 polymorphisms and cancer risk in humans across different countries [21]–[40]. However, these studies have appeared in the literature either supporting or negating the significant association. Some reviewed studies are limited by their sample size and subsequently suffer from too low power to detect effects that may exist. But the pool ORs generated from much larger population can increase the statistical power. Meta-analysis has great power for elucidating genetic factors in cancer. For better understanding of the association between this polymorphism and cancer risk, a pooled analysis with a large sample, subgroup analysis performed, and heterogeneity explored is necessary.

The results indicate that MTHFD1 G1958A and G401A are not risk factors for developing cancer in the overall study populations. The potential explanation is that the effect of a single polymorphism might have a limited impact on cancer susceptibility. This is in accordance with the hypothesis that cancer is a multifactorial disease that results from complex interactions between environmental and genetic factors. Nevertheless, considering that these 2 polymorphisms may play different roles in different cancer susceptibility among different ethnic subgroups and the frequencies of these 2 polymorphisms polymorphism might be different among different ethnic groups, we further conducted subgroup analysis by ethnicity and cancer type in current meta-analysis.

In this meta-analysis, when stratifying by ethnicity we found that the association between MTHFD1 G1958A polymorphism a decreased risk of cancer was significant only in Asians, not in the Caucasian population. Although the reasons for this difference remain controversial, there are several studies showing that it depends on a combination of differences in polymorphism distributions with nongenetic factors. Therefore, the difference of this polymorphism of MTHFD1 in Caucasians and Asians may result from interactions with environmental and social factors. On the other hand, the sample size and numbers of studies in Asian group were not adequate to evaluate the association. Other factors such as selection bias and different matching criteria may also play a role. In addition, considering the multistage character of cancer, genetic factors may play a role at specific stages only, which may vary between populations. Therefore, larger-scale studies and combined analysis are warranted to further confirm the effect of ethnic difference in this polymorphism on cancer risks. For G1958A, in the stratified analysis by cancer type, our results demonstrated that no significant associations were found in any genetic model among studies of colorectal cancer, head and neck cancer, and prostate cancer. For acute lymphoblastic leukemia, significantly decreased risk was observed in dominant model, and allelic model. With respect to other cancers, the results indicated that MTHFD1 G1958A was significantly associated with a decreased other cancers risk under recessive model. For G401A, MTHFD1 G401A polymorphism was associated with a decreased colon cancer risk under dominant model. One factor that would contribute to the discrepancy among different studies is that these 2 polymorphisms might play a different role in different cancer sites. However, even at the same cancer site, considering the possible small effect size of these 2 polymorphisms to cancer risk and the relatively small sample size in some studies, the discrepancy will become apparent since some of these studies may be underpowered to detect a small but real association. For acute lymphoblastic leukemia, there were only two studies included in the analysis with limited sample sizes, therefore, the results should be interpreted with caution. Considering the limited studies and population numbers of “other cancers” included in the meta-analysis, this may increase the risk of false negative findings. It is well recognized that there is individual susceptibility to the same kind of cancer even with the same environmental exposure. Host factors, including polymorphisms of genes involved in carcinogenesis, may have accounted for this difference. The discrepancy may have resulted from a differential effect of selection criteria in different cancers, which was dictated by the sample size in our meta-analysis, as well as the weight of each study. Other factors such as matched criteria may also have conferred an effect. The above differences may contribute to the inconsistent results. Therefore, it is very important to determine the unified selection criteria and to choose larger sample population studies.

Heterogeneity is a potential problem that might affect the interpretation of the results. Additionally, heterogeneity may influence the results of meta-analyses. For G401A, evident heterogeneity between studies was observed in overall comparisons and also in certain subgroup analyses. In overall analysis, significant between-study heterogeneity existed in allelic model comparison and dominant model comparison. After subgroup analyses by cancer type, the heterogeneity was removed in colon cancer. However, significant heterogeneity existed in allelic and dominant models when stratified according to cancer type. There are some factors that could have contributed toward the heterogeneity. First, the reason might be that different genetic backgrounds and the environment existed among different individuals. Second, one possibility involves differences in the matching status. However, we cannot confirm this possibility because no detailed information was provided. Third, we attempted to determine if the heterogeneity might also be explained by other variables such as smoking status, drinking status, and environmental factors included in the different studies, but are unable to provide a reliable answer to this question because we did not have access to individual level data for these variables.

Some limitations of this meta-analysis should be acknowledged. Firstly, only studies published were included in this meta-analysis, and nonsignificant or negative findings may be unpublished [44]–[46]. Hence, some inevitable publication biases might exist in the results. Secondly, controls were not uniformly defined. Healthy populations as well as non-cancer patients were included. Some individuals in the control group are likely to develop cancer in subsequent years though they had no clinical symptoms at the time of investigation. So selection bias may occur and they may not be representative of the general population. Thirdly, our result was based on unadjusted estimates, while a more precise analysis should be conducted adjusted by other factors like age, smoking status, drinking status, and environmental factors. Lack of information for data analysis may cause serious confounding bias. Fourthly, the meta-analysis was limited by a relatively small number of available studies. It is difficult to perform subgroup analysis for every type of cancer. Fifthly, in the subgroup analyses by ethnicity and cancer type, the sample size of studies among Asians and among several cancer types is small and limited, and the statistical power was so low that caution should be taken in interpreting these results. Sixthly, the association between G401A polymorphisms and ovarian cancer were based solely on the results of the Kelemen et al study which has been published previously, no additional information to the previously published study was found. In addition, lacking of the original data of the reviewed studies limited our further evaluation of potential interactions, because the interactions among gene-gene, gene-environment and even different polymorphic locis of the same gene may modulate cancer risk. Further investigations of the haplotypic effect of a gene and the study of multiple polymorphisms in different genes are needed.

### Conclusions

Our meta-analysis suggests that the MTHFD1 G1958A polymorphism might be associated with a decreased a decreased risk of ALL and other cancers. Meanwhile, the MTHFD1 G401A might play a protective role in the development of colon cancer. More well-designed epidemiological studies on specific ethnicity and cancer types, which were not well covered by existing studies, will be necessary to validate the findings identified in the current meta-analysis. Moreover, further evaluating the effect of gene–gene and gene–environment interactions on the MTHFD1 polymorphisms and cancer risk are necessary.

## Supporting Information

Figure S1 **The flow diagram for the review process and outcomes of inclusion and exclusion.**
(DOC)

Table S1 **Main characteristics of studies included in this meta-analysis.**
(DOC)
