# Supplementary material for: Association of Methylenetetrahydrofolate Dehydrogenase 1 Polymorphisms with Cancer: A Meta-Analysis
Source: PLoS One. 2013 Jul 19;8(7):e69366. doi: 10.1371/journal.pone.0069366 (PMC3716643; doi:10.1371/journal.pone.0069366)
Supplement: Figure S1 — The flow diagram for the review process and outcomes of inclusion and exclusion. (DOC) [file pone.0069366.s001.doc]

Manuscripts were excluded: Improper titles (n=16)

Potentially relevant manuscripts retrieved for abstract evaluation (n=47)

Manuscripts excluded after abstract review (n=8)

Potentially relevant manuscripts retrieved for detailed review (n=39)

20 eligible articles including 37 independent studies

17 studies were excluded due to:

Articles did not explore cancer risk (n=9)

Insufficient data for calculation of OR and 95%CI (n=5)

Duplicate data (n=2)

Review article (n=1)

Potentially relevant manuscripts were searched from Pubmed, Cochrane Library and Embase electronic databases up to February 20, 2013 (n=63)
