# Supplementary material for: Association of Methylenetetrahydrofolate Dehydrogenase 1 Polymorphisms with Cancer: A Meta-Analysis
Source: PLoS One. 2013 Jul 19;8(7):e69366. doi: 10.1371/journal.pone.0069366 (PMC3716643; doi:10.1371/journal.pone.0069366)
Supplement: Table S1 — Main characteristics of studies included in this meta-analysis. (DOC) [file pone.0069366.s002.doc]

**Table 1. Main characteristics of studies included in this meta-analysis**

| Study | Year | Ethnicity | Cases | | | | Controls | | | | HWE | Cancer type | Control source |
| --- | --- | --- | --- | --- | --- | --- | --- | --- | --- | --- | --- | --- | --- |
|  |  |  | GG | AG | | AA | GG | AG | | AA |  |  |  |
| R653Q, G1958A, rs2236225 | | |  |  | |  |  |  | |  |  |  |  |
| Liu et al. | 2012 | Caucasian | 448 | 716 | | 306 | 580 | 886 | | 370 |  | Colon cancer | PB |
| da Silva et al. | 2012 | Caucasian | 103 | 122 | | 47 | 179 | 235 | | 76 |  | Head/neck cancer | PB |
| Pawlik et al. | 2012 | Caucasian | 33 | 72 | | 30 | 39 | 85 | | 36 |  | Ovarian cancer | PB |
| Chan et al. | 2011 | Asian | 134 | 43 | | 8 | 115 | 53 | | 9 |  | ALL | - |
| Yang et al. | 2011 | Asian | 222 | 123 | | 16 | 200 | 151 | | 16 |  | ALL | PB |
| Mostowska et al. | 2011 | Caucasian | 41 | 61 | | 22 | 42 | 91 | | 35 |  | Cervical cancer | PB |
| Weiner et al. | 2011 | Caucasian | 44 | 79 | | 20 | 141 | 262 | | 136 |  | NHL | PB |
| Kruszyna et al. | 2010 | Caucasian | 41 | 57 | | 33 | 78 | 130 | | 42 |  | Laryngeal cancer | PB |
| Vaĭner et al. | 2010 | Caucasian | 240 | 443 | | 165 | 230 | 383 | | 171 |  | Breast cancer | PB |
| Collin et al. | 2009 | Caucasian | 496 | 774 | | 329 | 608 | 1042 | | 432 |  | Prostate cancer | PB |
| Murabito et al. | 2007 | Caucasian | 51 | 83 | | 38 | 66 | 117 | | 48 |  | Prostate cancer | PB |
| Stevens et al. | 2008 | Caucasian | 297 | 563 | | 238 | 311 | 560 | | 223 |  | Prostate cancer | PB |
| Yeager et al. | 2007 | Caucasian | 351 | 608 | | 204 | 348 | 548 | | 217 |  | Prostate cancer | PB |
| Eeles et al. | 2008 | Caucasian | 523 | 941 | | 387 | 543 | 958 | | 390 |  | Prostate cancer | PB |
| Amundadottir et al. | 2006 | Caucasian | 525 | 785 | | 300 | 10194 | 14639 | | 5697 | No | Prostate cancer | PB |
| Curtin et al. | 2007 | Caucasian | 314 | 436 | | 166 | 627 | 949 | | 396 |  | Colon cancer | PB |
| Chen et al. | 2004 | Caucasian | 90 | 128 | | 52 | 145 | 218 | | 85 |  | Colorectal cancer | HB |
| R134K, G401A, rs1950902 | | |  |  | |  |  |  | |  |  |  |  |
| Liu et al. | 2012 | Caucasian | 1023 | 396 | | 51 | 1213 | 560 | | 63 |  | Colon cancer | PB |
| Kelemen et al.-AUS | 2010 | Caucasian | 481 | 213 | | 22 | 712 | 320 | | 31 |  | Ovarian Cancer | PB |
| -DOV | 2010 | Caucasian | 331 | 178 | | 21 | 466 | 229 | | 23 |  | Ovarian Cancer | PB |
| -NCO1 | 2010 | Caucasian | 221 | 86 | | 6 | 294 | 144 | | 24 |  | Ovarian cancer | PB |
| -NCO2 | 2010 | Caucasian | 151 | 82 | | 12 | 184 | 64 | | 9 |  | Ovarian cancer | PB |
| -GER | 2010 | Caucasian | 129 | 62 | 6 | | 245 | 98 | 8 | |  | Ovarian cancer | PB |
| -HAW | 2010 | Caucasian | 46 | 22 | | 2 | 101 | 52 | | 4 |  | Ovarian cancer | PB |
| -HOP | 2010 | Caucasian | 206 | 71 | | 6 | 437 | 186 | | 16 |  | Ovarian cancer | PB |
| -MAL | 2010 | Caucasian | 280 | 121 | | 10 | 764 | 369 | | 49 |  | Ovarian cancer | PB |
| -MAY | 2010 | Caucasian | 187 | 109 | | 7 | 242 | 136 | | 10 |  | Ovarian cancer | HB |
| -NEC | 2010 | Caucasian | 224 | 80 | | 10 | 389 | 167 | | 13 |  | Ovarian cancer | PB |
| -NHS | 2010 | Caucasian | 74 | 29 | | 2 | 220 | 119 | | 13 |  | Ovarian cancer | PB |
| -SEA | 2010 | Caucasian | 341 | 163 | | 24 | 803 | 361 | | 54 |  | Ovarian cancer | PB |
| -STA | 2010 | Caucasian | 165 | 74 | | 9 | 251 | 101 | | 11 |  | Ovarian cancer | PB, FB |
| -UCI | 2010 | Caucasian | 197 | 73 | | 11 | 283 | 131 | | 13 |  | Ovarian cancer | PB |
| -UKO | 2010 | Caucasian | 169 | 78 | | 7 | 376 | 171 | | 24 |  | Ovarian cancer | PB |
| -USC | 2010 | Caucasian | 283 | 102 | | 12 | 381 | 163 | | 15 |  | Ovarian cancer | PB |
| Stevens et al. | 2007 | Caucasian | 316 | 163 | | 13 | 353 | 124 | | 16 |  | Breast cancer | PB |
| Curtin et al. | 2007 | Caucasian | 624 | 265 | | 27 | 1319 | 585 | | 68 |  | Colon cancer | PB |
| Metayer et al. | 2011 | Caucasian | 263 | 98 | | 12 | 302 | 124 | | 17 |  | ALL | PB |

HWE, Hardy-Weinberg equilibrium; HB, hospital based; PB, population based; FB, family based; NHL, non-Hodgkin’s lymphoma; ALL, acute lymphoblastic leukemia
